# Cost–utility analysis of imatinib mesilate for the treatment of advanced stage chronic myeloid leukaemia

**DOI:** 10.1038/sj.bjc.6601151

**Published:** 2003-08-12

**Authors:** A Gordois, P Scuffham, E Warren, S Ward

**Affiliations:** 1 York Health Economics Consortium Ltd, University of York, Market Square (level 2), York YO10 5NH, UK; 2 School of Health and Related Research, University of Sheffield, Regent Court, 30 Regent Street, Sheffield S1 4DA, UK

**Keywords:** chemotherapy, cost-effectiveness, economic evaluation, Glivec

## Abstract

Imatinib mesilate (Glivec®, Novartis Pharmaceuticals) is a novel therapy for the treatment of chronic myeloid leukaemia (CML). We evaluated the cost-effectiveness of imatinib (600 mg daily) when used for the treatment of patients in advanced stages of CML (accelerated phase and blast crisis) against conventional therapies of combination chemotherapy (DAT) and palliative care in hospital or at home. A Markov model simulated the transitions of hypothetical patient cohorts and outcomes were modelled for 5 years from the start of treatment. Costs were estimated from the perspective of the UK National Health Service. Over 5 years, a patient in accelerated phase will, on average, accrue an additional 2.09 QALYs with imatinib compared to conventional therapies, while patients in blast crisis will accrue an additional 0.58 quality-adjusted life-years (QALYs) with imatinib compared to conventional therapies. The costs per additional QALY gained from treatment with imatinib compared with conventional therapies were £29 344 (accelerated phase) and £42 239 (blast crisis). The results were particularly sensitive to the price of imatinib, improvements in quality of life, and the duration of haematological responses. We conclude that treatment of CML with imatinib confers considerably greater survival and quality of life than conventional treatments but at a cost.

Chronic myeloid leukaemia (CML) accounts for around 15–20% of all adult leukaemias (O'Brien, 2000). The incidence of CML is 1.0–1.5 new cases per 100 000 population (Kolibaba and Druker, 2000; O’Brien, 2000). In the UK, there are approximately 3000 patients with CML and 600–800 new cases are diagnosed annually (O’Brien, 2000). The median age at diagnosis of CML is approximately 50 years; it is relatively uncommon in people under the age of 20 years, and is more common among male subjects (Bogard and Scheer, 2001).

If untreated, a patient will remain in the chronic phase of CML for a median of 4–5 years before progressing to an acute and fatal blast crisis phase. A transitional accelerated phase, lasting 3–9 months, is experienced by 60–80% of patients. The median patient survival is only 3–6 months in blast crisis (Sawyers, 1999; Kolibaba and Druker, 2000).

Reducing the numbers of leucocytes in the bloodstream (haematological responses) and Philadelphia-positive (Ph+) cells in the bone marrow (cytogenetic responses) is the therapeutic aim. Reductions in Ph+ metaphases to less than 35% of the total (major cytogenetic responses) have been associated with long-term survival (The Italian Cooperative Study Group on Chronic Myeloid Leukemia, 1994; Allan *et al*, 1995; Kantarjian *et al*, 1995). However, therapeutic efficacy in the accelerated phase and blast crisis is poor. Bone marrow transplantation (BMT) is the only potentially curative therapy as conventional therapies (eg *α*-interferon, hydroxyurea) do not to lead to long-term disease-free survival. Generally, patients in advanced stages of CML receive palliative care or experimental chemotherapy (Bogard and Scheer, 2001; Kolibaba and Druker, 2000). However, younger patients might receive intensive chemotherapy or BMT.

Imatinib mesilate (Glivec®, Novartis Pharmaceuticals) is an orally administered treatment specifically targeting cancerous cells (Druker *et al*, 2001a,2001b). Phase II trial results show relatively high haematological and cytogenetic response rates (Kantarjian *et al*, 2002, Sawyers *et al*, 2002), even in the accelerated and blast phases where prognosis is usually poor (Capdeville and Gathmann, 2001). Although the duration of follow-up is currently too short to assess long-term survival, indications suggest that imatinib offers a considerable survival advantage over chemotherapy and palliative care.

Imatinib's potential benefits, improved survival and quality of life, may be countered by increased patient management costs. This study evaluates the cost-effectiveness of imatinib when used in the accelerated and blast crisis phases of CML compared with conventional therapies. Using a Markov model, we estimated the direct costs to the UK National Health Service (NHS) of these additional health gains.

## MATERIALS AND METHODS

The analysis was undertaken for four patient cohorts: patients presenting in accelerated phase and treated with imatinib (the accelerated phase study cohort), patients presenting in accelerated phase and treated with conventional therapies (the accelerated phase comparator cohort), patients presenting in blast crisis and treated with imatinib (the blast crisis study cohort), and patients presenting in blast crisis and treated with conventional therapies (the blast crisis comparator cohort). We compared the costs and consequences for the accelerated phase study and comparator cohorts, and for the blast crisis study and comparator cohorts. The total health care costs were calculated from the average patient management costs and the number of patients in each health state. Utility values for patient quality of life were assigned to each health state and treatment regimen. Total quality-adjusted survival and costs were calculated over a simulated period of 5 years.

### Markov model

A Markov model was developed to simulate the transitions of a hypothetical cohort of 1000 CML patients from the point at which they present for treatment, through a series of health states to death (Beck and Pauker, 1983; Sonnenberg and Beck, 1993). Markov models are practical tools for modelling chronic diseases. In each 1-month cycle, patient's movement between health states is determined by transition probabilities.

**Figure 1:**
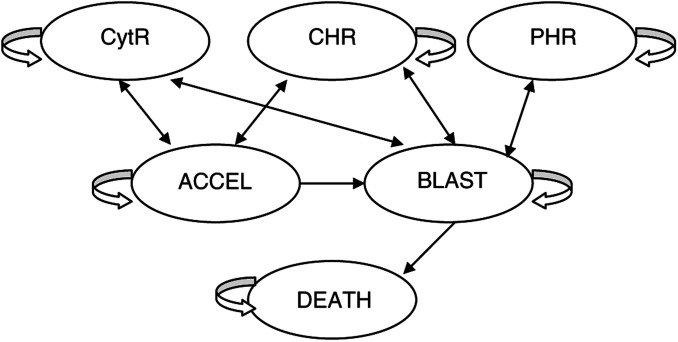
Markov model process depicting movement between health states.

Patients are either newly diagnosed with CML in accelerated phase or blast crisis or have previously failed conventional treatment in chronic phase, by progressing to the advanced disease stage. The health states in our model represent the major clinical end points in randomised controlled trials, and correspond to previous cost–utility analyses (Kattan *et al*, 1996; Liberato *et al*, 1997). The model is depicted in Figure 1

.

Patients in the accelerated phase (ACCEL) or blast crisis (BLAST) may respond to imatinib, having either a complete haematological response accompanied by a major cytogenetic response (CytR: a return to normal white blood cell (WBC) counts and a reduction in Ph^+^ metaphases to ⩽35% of the total), complete haematological response without major cytogenetic response (CHR: a return to normal WBC counts with no major reduction in Ph^+^ metaphases), or partial haematological response (PHR: the WBC count falls but is still above the normal level). Patients remain in response states until progressing to ACCEL or BLAST. Therefore, these responses can be considered the ‘best response’. Unresponsive patients progress from ACCEL to BLAST or DEATH, and from BLAST to DEATH. In all health states, patients may die from disease-unrelated causes.

### Treatments

Accelerated phase and blast crisis study cohorts receive 600 mg of imatinib per day at home. On failing imatinib (progressing from ACCEL to BLAST or losing their response), patients switch to conventional therapies: combination chemotherapy or palliative care. Combination chemotherapy (DAT) is comprised of one course of daunorubicin, cytarabine arabinoside (Ara-C) and 6-Tioguanine, given in hospital as an in-patient. Palliative care may be provided at hospital or in the patient's home. The costs for palliative care treatments are not specified in this study because expenditure is either absorbed within hospital bed-day costs or incurred by the caregiver at no cost to the NHS. In the absence of imatinib (the comparator cohorts) patients receive conventional therapy from the outset.

The possibility that patients undergo BMT is excluded from the analysis. Patients eligible for BMT are likely to have undergone the procedure immediately on failure of front-line treatment (i.e., on progressing to the accelerated phase or blast crisis from the chronic phase). The proportion of patients newly diagnosed in the advanced stages who undergo BMT is minimal. Additionally, it is unclear whether BMT is indicated if the patient responds to, or is resistant to, imatinib.

### Probabilities

Transition probabilities for the accelerated phase and blast crisis study cohorts were obtained from the international Phase II imatinib clinical trials 0109 (237 patients presenting in accelerated phase CML; Kantarjian *et al*, 2002) and 0102 (260 patients presenting in blast crisis CML; Sawyers *et al*, 2002; Capdeville and Gathmann, 2001).

Probabilities for the accelerated phase cohort were derived from the clinical trial for patients receiving 600 mg imatinib per day (158 patients). Probabilities for the blast crisis cohort were derived from the trial for patients who were newly diagnosed with blast crisis and, therefore, not previously treated for that disease stage (165 patients). For accelerated-phase patients, data had not been disaggregated by prior treatment, and for blast crisis patients, data had not been disaggregated by imatinib daily dose. We excluded the data on patients who were not assessable.

To calculate transition probabilities we estimated the rates of response, disease progression and death in nonresponders, and disease progression for those who had initially responded to treatment but their response had waned. It was assumed that all major cytogenetic responses accompanied a CHR (Kantarjian *et al*, 1995; Kattan *et al*, 1996; Liberato *et al*, 1997). The rates of CHR (i.e., without major cytogenetic response) were estimated as the trial rates minus the rate of major cytogenetic response. In trial 0102, the rate of major cytogenetic response exceeded the rate of CHR. We assumed the remaining major cytogenetic responses accompanied a PHR, and the rate of PHR was reduced accordingly (Table 1

**Table 1:** Model disease progression rates

**Treatment**	**From health state**	**To health state**	**Rate^a^**	**Reference**
Imatinib mesilate	ACCEL	CytR	28.6%	
	ACCEL	CHR	12.2%	
	ACCEL	PHR	34.0%	
	ACCEL	BLAST^b^	10.9%	
	ACCEL	DEATH^c^	22.0% (95% CI: 15.0–29.0%)	(Capdeville and Gathmann, 2001)
	ACCEL - CytR/CHR/PHR	ACCEL or BLAST^d^	22.5% (95% CI: 13.6–31.5%)	
	BLAST	CytR	15.7%	
	BLAST	CHR	0.0%	
	BLAST	PHR	20.9%	
	BLAST	DEATH^c^	67.9% (95% CI: 56.8–79.0%)	
	BLAST - CytR/CHR/PHR	ACCEL or BLAST^d^	46.1% (95% CI: 30.9–61.3%)	
				
Palliative care/DAT	ACCEL	BLAST	50% at 6 months	(Kolibaba and Druker, 2000; Reese *et al*, 2000)
	BLAST	DEATH	50% at 4.5 months	
				
Disease-unrelated death	Any	DEATH	0.04%	(Kattan *et al*, 1996)

a Rates at 12 months, 9 months (CytR/CHR/PHR to BLAST) and 1 month (disease-unrelated death).

b Progression without response (trial 0109).

c Overall survival (trials 0109 and 0102).

d Inverse of haematological response duration rate (trials 0109 and 0102).

).

All rates were converted to monthly probabilities (Beck and Pauker, 1983; Sonnenberg and Beck, 1993). As a proportion of patients’ progress to blast crisis without an intervening accelerated phase, the probability for loss of response was weighted between accelerated phase (70%) and blast crisis (30%). For the comparator cohorts, monthly transition probabilities were calculated from the median durations of accelerated phase and blast crisis (Kolibaba and Druker, 2000; Reese *et al*, 2000). It was assumed that, on average, DAT does not confer greater survival relative to palliative care.

A clinician panel estimated the proportions of newly diagnosed patients in the UK assigned to each comparator therapy (DAT, hospital palliative care or home palliative care). The clinician panel also estimated the proportions for accelerated phase patients progressing to blast crisis for each treatment arm (Table 2

**Table 2:** Proportions of patients receiving each treatment

	**Accelerated Phase**				**Blast crisis**	
**Health states**	**DAT (%)**	**Hospital palliative care (%)**	**Home palliative care (%)**	**DAT (%)**	**Hospital palliative care (%)**	**Home palliative care (%)**
Newly diagnosed	10	0	90	60	30	10
Chronic phase treatment failure or progression of disease from CytR/CHR/PHR (Imatinib)	10	0	90	50	10	40
*Previously treated in accelerated phase with*						
DAT	N/A	N/A	N/A	30	20	50
Hospital palliative care	N/A	N/A	N/A	50	10	40
Home palliative care	N/A	N/A	N/A	45	21	34

).

### Quality of life

The outcomes used in this study were quality-adjusted life-years (QALYs). Patient's quality of life, for each treatment and health state, was assessed using the ‘self-reported description’ component of the EuroQol (EQ-5D) instrument. Five dimensions are assessed (mobility, self-care, performance of usual activities, pain/discomfort and anxiety/depression), each having three possible responses (‘no problem’, ‘some difficulties/moderate problem’ or ‘unable/extreme problem’). Each combination of responses has an associated utility value derived from a representative sample of the UK public, indexed between 0 (dead) and 1 (full health); health states with negative utilities are valued worse than death.

In the absence of utility data associated with imatinib, DAT or palliative care, a clinician panel as used to estimate the quality of life for an average patient in each health state and treatment subgroup; the means of clinician responses (six respondents) were used in the model (Table 3

**Table 3:** Mean (range) of utility values for health states and treatments

**Health state**	**Imatinib**	**Combination chemotherapy**	**Hospital palliative care**	**Home palliative care**
CytR	0.91 (0.73–1.00)	N/A	N/A	N/A
CHR	0.91 (0.73–1.00)	N/A	N/A	N/A
PHR	0.91 (0.73–1.00)	N/A	N/A	N/A
ACCEL	0.58 (0.15–1.00)	0.01 (−0.33 to 0.52)	0.07 (−0.33 to 0.52)	0.34 (0.08 to 0.52)
BLAST	0.38 (0.02–0.69)	−0.09 (−0.33 to 0.52)	−0.18 (−0.33 to 0.02)	0.04 (−0.17 to 0.20)
DEATH	0.00	0.00	0.00	0.00

NA=not applicable.

). Clinician-reported utilities might be less representative of patient's quality of life than if the scores were obtained from a large patient sample. Therefore, subsequent benefit and cost-effectiveness results may be underestimated or overestimated.

QALYs were calculated for each monthly cycle as the number of patients in each health state and treatment subgroup multiplied by their utility values. QALYs were discounted at an annual rate of 1.5% following the NICE recommendations (National Institute for Clinical Excellence, 2001). Discounted QALYs accrued in each cycle were summed over the 5 years to determine the total discounted QALYs amassed by the cohort.

### Costs and resource use

Direct costs incurred by the NHS were included in the analysis (Table 4

**Table 4:** Unit costs of resources

**Resource**	**Unit cost**	**Reference**
Imatinib	£90.00 per day	*Choice parameter*
Combination chemotherapy (drugs)	£575.24 per course^a^	The Chartered Institute of Public Finance and Accountancy (CIPFA) (2000) and British National Formulary (2001)
Combination chemotherapy (bed days)	£5068.00 per course	The Chartered Institute of Public Finance and Accountancy (CIPFA) (2000)^b^
Hospital palliative care	£181.00 per day	The Chartered Institute of Public Finance and Accountancy (CIPFA) (2000)^b^
Blood transfusions	£3242.70 per episode^c^	National Blood Donor Registry: Leeds, UK, (Netten *et al*, 2002)
Outpatient clinic visit/ bone marrow test	£60.00 per visit/test	Department of Health (2002)^d^
Chest X-ray	£21.00 per X-ray	Personal communication (2001)^e^
CT scan	£151.00 per scan	Personal communication (2001)^e^
Nurse home visit	£19.00 per visit	Netten *et al* (2002)
GP home visit	£45.00 per visit	Netten *et al* (2002)

a Based on daily doses of daunorubicin (50 mg m^−2^ for 3 days), Ara-C (100 mg m^−2^ twice for 10 days) and 6-Tioguanine (2.5 mg kg^−1^ for 10 days), and costs per course of £365.66 (daunorubicin), £131.89 (Ara-C) and £77.68 (6-Tioguanine). The mean patient body surface area and weight of 1.74 m^2^ and 70 kg are assumed (Smithies, 1996; Gorin, 1998; Lee *et al*, 1998; Messori, 1998).

b Other Medicine, all English Trusts.

c Based on 20 U of full blood (£82.70 U^−1^), 10 U of platelets (£151.27 U^−1^) and 2 h nursing time (£38.00 h^−1^) per accelerated phase/blast crisis.

d Follow-up clinical haematology outpatient attendance.

e Personal communication with six NHS Trusts (trimmed mean of responses). Further details available from the authors on request.

). Consumption taxes, known in the UK as VAT, were excluded from all costs. VAT is a transfer payment from one sector in the economy to another, and therefore, there is no net cost or gain to the government.

Drug costs were the averages of those listed in the British National Formulary (2001). Imatinib's price is £12.98 per 100 mg. Patients receiving imatinib attend an outpatient clinic fortnightly in accelerated phase or weekly in blast crisis. In response states, patients are assumed to attend, on average, once every 8 weeks until disease progression.

For patients receiving DAT, their length of stay was assumed to be 28 days. For patients receiving hospital palliative care, we assumed a hospital stay of 1 month, or, for those who switched to home palliative care or died during the monthly cycle, a hospital stay of 0.5 months was assumed. The bed-day cost was assumed to exclude intensive care but include palliative treatments. Patients receiving DAT incur the cost of one course of DAT in addition to hospital bed-day costs. Home palliative care was assumed to place no cost on the NHS.

We assumed that patients assigned to imatinib or DAT undergo a bone marrow test on disease progression, and patients receiving imatinib undergo an additional bone marrow test every 6 months (including those in response states). Palliative care patients receive no examinations since their therapies are not aimed at response inducement. Since data were unavailable on the cost of a bone marrow examination, we assumed the cost was equal to that of an outpatient appointment. Full blood counts, blood chemistry, and physical examination are routine, low-cost tests and their cost is absorbed within outpatient visit costs.

During blast crisis, all patients receive three chest X-rays (on average) and 20% receive a CT scan. Patients receiving home palliative care are not tested, but patients assigned to DAT or hospital palliative care receive these examinations during the inpatient period. Therefore, only the test costs themselves are included in the analysis. During the accelerated phase or blast crisis, patients may also receive blood transfusions. It cannot be determined when these tests and transfusions occur, but only that, on average, the costs are incurred during the disease stage. In our model, these costs of tests and transfusions were attributed to the patient in the first month of accelerated phase or blast crisis.

All patients, except hospital inpatients, receive a district nurse home visit twice per month. Those receiving palliative care are also visited by their GP once a month. The costs associated with these home visits were included for all cohorts.

The total costs were estimated for all patients in each health state, in each monthly cycle. Costs were discounted at an annual discount rate of 6%, in line with the NICE recommendations (National Institute for Clinical Excellence, 2001). A 5-year total cost of treatment was then estimated for each cohort.

### Incremental cost-effectiveness ratios

An incremental cost-effectiveness ratio (ICER) is calculated as the ratio of the difference in total QALYs to the difference in total costs between the study and comparator cohorts:


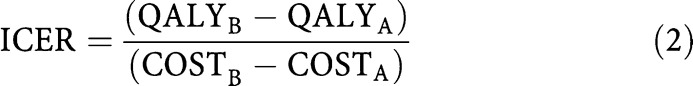


where QALY is the total QALYs, COST=total costs, A the comparator cohort, and B=study cohort. The ICER is the additional cost per additional QALY gained from treatment with imatinib.

### Sensitivity analysis

Parameters where there was the greatest uncertainty, and to which imatinib's cost-effectiveness was expected to be particularly sensitive, were varied within plausible ranges (Table 5

**Table 5:** Parameters tested in the sensitivity analysis

**Parameter**	**Baseline estimate**	**Range**
Imatinib price per 100 mg	£12.98	£6.49–£12.98^a^
12-month rate of response loss (accelerated phase study cohort)	22.5%	13.6–31.5%^b^
Nine-monthly rate of response loss (blast crisis study cohort)	46.1%	30.9–61.3%^c^
Utility of imatinib in CytR/CHR/PHR	0.91	0.73–1.00^d^
Utility of imatinib in ACCEL	0.58	0.15–1.00^d^
Utility of imatinib in BLAST	0.38	0.02–0.74^d^
Discount rate for QALYs	1.5%	0–6^e^
Discount rate for costs	6%	0–10^e^

a A maximum price discount of 50% might be possible in the long-term future.

b Based on 95% confidence interval for 12-month duration of haematological response (trial 0109).

c Based on 95% confidence interval for 9-month duration of haematological response (trial 0102).

d Range of estimates from clinician panel.

e National Institute for Clinical Excellence, 2001.

). These parameters included the acquisition cost (price) of imatinib, survival benefits, utility values, and the discount rates for costs and benefits. ICERs were also estimated for best- and worst-case scenarios by setting these parameters at the most optimistic or pessimistic limit in their range.

## RESULTS

For the accelerated phase study cohort, 1-year survival and progression-free survival rates of 80.1 and 75.0%, respectively, were estimated from the model. These are substantially greater than the estimates (54.8 and 24.9%) obtained for the accelerated phase comparator cohort where patients receive DAT followed by home palliative care (10% patients) or home palliative care only (90% patients). From the model, we estimated median survivals of 39.5 months (study cohort) and 13.1 months (comparator cohort). In the study cohort, the 5-year per-patient costs to the NHS were £61 268 greater and each patient, on average, accrued 2.09 additional QALYs compared to the comparator cohort. The ICER for the accelerated phase was £29 344 per QALY.

For the blast crisis study cohort, a 1-year survival rate of 40.4% was estimated from the model. This is more than twice the survival rate (15.7%) estimated for the comparator cohort where patients receive DAT followed by home palliative care (51.4% patients), hospital palliative care (12.9% patients), or home palliative care only (35.7% patients). From the model, we estimated median survivals of 8.7 months (study cohort) and 4.5 months (comparator cohort). In the study cohort, the 5-year per-patient costs to the NHS were £24 695 greater and each patient, on average, accrued 0.58 additional QALYs compared to the comparator cohort. The ICER for blast crisis was £42 239 per QALY (Table 6

**Table 6:** Results of the baseline analysis (per patient)

	**Imatinib**	**Comparator**	**Incremental costs and QALYs**
*Accelerated phase (1000 patients)*			
Total discounted cost	£78 593	£17 325	£61 268
Total discounted QALYs	2.04	−0.04	2.09
ICER (£ per additional QALY)			£29 344
			
*Blast crisis (1000 patients)*			
Total discounted Cost	£35 781	£11 085	£24 695
Total discounted QALYs	0.53	−0.05	0.58
ICER (£ per additional QALY)			£42 239

).

The incremental costs and QALYs both decreased each year over the 5 years modelled (Table 7

**Table 7:** Annual incremental costs and QALYs (per patient)

	**Accelerated phase**		**Blast crisis**	
	**Incremental**	**Incremental**	**Incremental**	**Incremental**
**Year**	**costs**	**QALYs**	**costs**	**QALYs**
1	£18 456	0.65	£13 239	0.27
2	£14 327	0.51	£4528	0.09
3	£12 084	0.40	£1601	0.02
4	£9391	0.30	£565	0.01
5	£7010	0.23	£194	0.00

). For initial cohorts of 1000 patients, the average incremental cost per patient in year 5 was 38% of the year 1 cost for patients in accelerated phase and 4.1% for patients in blast crisis. These different annual costs reflect different survival rates for the different stages of CML. The incremental costs per QALY increased over time for both accelerated phase (trend = £245 per month) and blast crisis (trend = £65 per month).

### Sensitivity analysis

Imatinib's cost-effectiveness was sensitive to its price (Table 8

**Table 8:** Results of the sensitivity analysis (per patient)

	**Accelerated phase**			**Blast crisis**		
**Parameter/value**	**Incremental costs**	**Incremental QALYs**	**ICER**	**Incremental costs**	**Incremental QALYs**	**ICER**
Baseline^a^	£61 268	2.09	£29 344	£24 695	0.58	£42 239
*Imatinib price per 100 mg*						
£6.49 (50% discount)	£27 719	2.09	£13 276	£11 237	0.58	£19 220
£7.79 (40% discount)	£34 439	2.09	£16 495	£13 929	0.58	£23 824
£9.09 (30% discount)	£41 159	2.09	£19 713	£16 620	0.58	£28 428
£10.38 (20% discount)	£47 828	2.09	£22 907	£19 312	0.58	£33 032
£11.68 (10% discount)	£54 548	2.09	£25 126	£22 004	0.58	£37 636
						
*Rates of response loss*^b^						
13.6% (response achieved from ACCEL)						
30.9% (response achieved from BLAST)	£69 028	2.44	£28 308	£28 212	0.73	£38 766
31.5% (response achieved from ACCEL)						
61.3% (response achieved from BLAST)	£54 606	1.80	£30 417	£22 293	0.49	£45 274
						
*Imatinib utility values*						
0.73 (CytR/CHR/PHR)						
0.15 (ACCEL)						
0.02 (BLAST)	£61 268	1.43	£42 958	£24 695	0.29	£85 981
1.00 (CytR/CHR/PHR)						
1.00 (ACCEL)						
0.74 (BLAST)	£61 268	2.58	£23 717	£24 695	0.85	£28 937
						
*Discount rates*						
0% (costs), 0% (QALYs)	£69 528	2.15	£32 302	£26 654	0.59	£44 853
6% (costs), 0% (QALYs)	£61 268	2.15	£28 465	£24 695	0.58	£41 556
6% (costs), 6% (QALYs)	£61 268	1.91	£32 060	£24 695	0.56	£44 291
10% (costs), 0% (QALYs)	£56 555	2.15	£26 275	£23 526	0.59	£39 589
10% (costs), 10% (QALYs)	£56 555	1.77	£31 907	£23 526	0.54	£43 932
						
*Extreme scenarios*						
Worst case^c^	£61 388	1.01	£60 991	£23 838	0.20	£122 016
Best case^d^	£27 959	3.06	£9132	£11 897	1.03	£11 556

a Imatinib price per 100 mg=£12.98, monthly probability of response loss (response achieved from ACCEL)=0.0210, monthly probability of response loss (response achieved from BLAST) =0.0664, Imatinib utility (CytR/CHR/PHR)=0.91, Imatinib utility (ACCEL)=0.58, Imatinib utility (BLAST)=0.38, discount rate (costs)=6%, discount rate (QALYs)=1.5%.

b At 12 months (responses achieved from ACCEL) and nine months (responses achieved from BLAST).

c Imatinib price per 100 mg=£12.98, monthly probability of response loss (response achieved from ACCEL)=0.0310, monthly probability of response loss (response achieved from BLAST)=0.1001, Imatinib utility (CytR/CHR/PHR)=0.73, Imatinib utility (ACCEL)=0.15, Imatinib utility (BLAST)=0.02, discount rate (costs)=0%, discount rate (QALYs)=10%.

d Imatinib price per 100 mg=£6.49, monthly probability of response loss (response achieved from ACCEL)=0.0121, monthly probability of response loss (response achieved from BLAST)=0.0402, Imatinib utility (CytR/CHR/PHR)=1.00, Imatinib utility (ACCEL)=1.00, Imatinib utility (BLAST)=0.74, discount rate (costs)=10%, discount rate (QALYs)=0%.

). For all discounted costs within the range, treatment with imatinib incurred additional costs compared with conventional treatments. If the price of imatinib was discounted by 50% (£6.49 per 100 mg), the ICERs were 54% lower.

Since the confidence interval of 12-month haematological response loss from imatinib in accelerated phase was narrow, imatinib's cost-effectiveness was insensitive to duration of response. In blast crisis, where confidence intervals were wider, the ICER was up to 7 higher or 8% lower.

Imatinib's cost-effectiveness was sensitive to patient utility values. In the accelerated phase, the ICER was up to 46% higher or 19% lower. In blast crisis, the ICER was up to 104% higher or 31% lower. The choice of discount rates used had a relatively small effect on the cost-effectiveness ratios.

Under a best-case scenario, the ICERs were 69% lower (accelerated phase) and 43% lower (blast crisis). Under a worst-case scenario, the costs per QALY were substantially greater than at baseline (108% higher in accelerated phase; 189% higher in blast crisis).

## DISCUSSION

There have been no studies previously published which evaluate the cost-effectiveness of treatments in the advanced stages of CML. This may be due to the similarity in profiles between conventional palliative therapies, inducing only temporary and/or partial responses and rarely offering improved survival. Imatinib offers considerable advantages over current treatments in both survival and quality of life for the advanced stages of CML. However, the monthly drug cost is high compared with conventional therapies (eg low-cost home palliative care). We estimated that the marginal cost of treatment with imatinib was £29 000 (accelerated phase) and £42 000 (blast crisis) per QALY over a 5-year period. Imatinib was more cost-effective in the accelerated phase due to considerably better patient survival and quality of life. After 12 months of treatment, 78% of patients in accelerated phase survived (trial 0109, 600 mg dose group) compared with 32% survival in blast crisis (trial 0102, previously untreated group) (Capdeville and Gathmann, 2001). Moreover, 67% of accelerated phase patients had not experienced disease progression at 12 months.

There is uncertainty surrounding the duration of response from imatinib during blast crisis. However, varying this parameter had little effect on imatinib's cost-effectiveness. Using 95% confidence intervals of response duration, the cost per QALY are up to 7% higher (shortest duration) or 8% lower (longest duration) than the baseline estimate. For longer responses, the increase in QALYs outweighed the additional expense of imatinib treatment resulting in a lower ICER.

Imatinib responders have a substantially better quality of life than patients receiving current treatments, even under pessimistic assumptions. The lowest estimated utility value with imatinib for unresponsive blast crisis patients (0.02) is similar to the baseline utility value for home palliative care (0.04) and greater than the baseline utility value for hospital palliative care (−0.18). Imatinib's cost-effectiveness in blast crisis varied considerably over the range of utility values. At lower utility values, health benefits are overshadowed by the drug price and the ICER is substantially higher.

There are several qualifications to our model. However, our approach to this analysis was to favour the comparator wherever there was uncertainty. As the Phase II trials were one-arm trials it was not possible to elicit controlled utility values from patients. In the absence of primary patient data, we resorted to using a clinician panel. The EQ-5D responses elicited from the clinician panel may possibly reflect the average patient's quality of life more accurately than those elicited from a patient sample. We expect clinicians to have greater experience of CML patients than an individual with the disease. Conversely, clinicians’ ‘objective’ assessments may be no substitute for patients’ experiences. Therefore, individual patients may convey the disease's impact more accurately than that perceived by clinicians. These issues need to be addressed in future research.

We assumed that a full month of imatinib treatment was given in each cycle. This should not substantially overstate the duration of treatment with imatinib since there is likely to be a lag between disease progression (treatment failure) and clinical confirmation (cessation of treatment). As imatinib is considerably more expensive than palliative care, this bias favours the comparator.

Patients may, in reality, move between response states (e.g., a partial response may be attained prior to a complete response). Owing to limitations in data availability we excluded movement between response states representing ‘best responses’. This limits the accuracy of our overall and progression-free survival estimates, and may underestimate the effectiveness of imatinib.

We modelled outcomes for 5 years. The relative magnitudes of incremental costs and benefits determine imatinib's cost-effectiveness over longer time horizons. Additional data on the long-term effectiveness, changes in response, quality of life and contraindications of imatinib are required over the longer-term to validate our modelled effectiveness and cost estimates. In particular, the duration of follow-up in the clinical trials as too short to assess long-term survival. Actual 5-year survival rates with imatinib may be substantially lower than those estimated by assuming a constant monthly probability of death.

The effectiveness data used for this study was obtained from the manufacturer of Imatinib (Novartis). These data are reported elsewhere (Capdeville and Gathmann, 2001, Kantarjian *et al*, 2002; Sawyers *et al*, 2002). In addition, these data, this cost-effectiveness model and the results have been reviewed by the NICE. Therefore, any potential bias from the source of funding for this study (i.e., Novartis) should be minimal. Moreover, we have used conservative assumptions for any parameters where there was uncertainty; this will bias results in favour of the comparators rather than imatinib.

The impact on NHS budgets in switching from conventional practice to treatment with imatinib for patients in the advanced stages of CML is a major policy consideration. Assuming 70% of advanced stage CML patients are in accelerated phase and 30% in blast crisis, the costs per patient for treatment with imatinib in years 1 and 5 were £17 300 and £5100, respectively. For an estimated 1000 advanced stage CML patients in the UK, the average cost to each of the 28 new strategic health authorities (StHAs) will be approximately £0.6 m per year. This cost will have a relatively small impact on the budgets of StHAs; and given that the alternative treatments for CML are considerably less effective, imatinib mesilate is the logical and economically feasible treatment for those with advanced stages of CML.

## References

[bib1] Allan NC, Richards SM, Shepherd PC (1995) UK Medical Research Council randomised, multicentre trial of interferon-alpha n1 for chronic myeloid leukaemia: improved survival irrespective of cytogenetic response. The UK Medical Research Council's Working Parties for Therapeutic Trials in Adult Leukaemia. Lancet 345: 1392–1397 7760609 10.1016/s0140-6736(95)92596-1

[bib2] Beck JR, Pauker SG (1983) The Markov process in medical prognosis. Med Decis Making 3: 419–458 6668990 10.1177/0272989X8300300403

[bib3] Bogard B, Scheer D (2001) Imatinib STI571: A Recommended Pharmacoeconomic Strategy, HPM201b Final Project, Camberley: Novartis Pharma

[bib4] British National Formulary (2001) BNF 41 March 2001. London: British Medical Association and the Royal Pharmaceutical Society of Great Britain

[bib5] Capdeville R, Gathmann I (2001) Integrated summary of efficacy–Update (cut-off 31-Jan-01). Basel: Novartis Pharma AG

[bib6] Department of Health (2002) NHS Reference Costs 2001. London: Department of Health

[bib7] Druker BJ, Talpaz M, Resta DJ, Peng B, Buchdunger E, Ford JM, Lydon NB, Kantarjian H, Capdeville R, Ohno-Jones S, Sawyers CL (2001a) Efficacy and safety of a specific inhibitor of the BCR-ABL tyrosine kinase in chronic myeloid leukemia. N Engl J Med 344 (14): 1031–1037 11287972 10.1056/NEJM200104053441401

[bib8] Druker BJ, Sawyers CL, Kantarjian H, Resta DJ, Reese SF, Ford JM, Capdeville R, Talpaz M (2001b) Activity of a specific inhibitor of the BCR-ABL tyrosine kinase in the blast crisis of chronic myeloid leukemia and acute lymphoblastic leukemia with the Philadelphia chromosome. N Engl J Med 344 (14): 1038–1042 11287973 10.1056/NEJM200104053441402

[bib9] Gorin NC (1998) Autologous stem cell transplantation in acute myelocytic leukemia. Blood 92: 1073–1090 9694694

[bib10] Kantarjian HM, Smith TL, O’Brien S, Beran M, Pierce S., Talpaz M. (1995) Prolonged survival in chronic myelogenous leukemia after cytogenetic response to interferon-alpha therapy. The Leukemia Service. Ann Intern Med 122: 254–261 7825760 10.7326/0003-4819-122-4-199502150-00003

[bib11] Kantarjian HM, O’Brien S, Cortes JE, Smith TL, Rios MB, Shan J, Yang Y, Giles FL, Thomas DA, Faderl S, Garcia-Manero G, Jeha S, Wierda W, Issa JP, Kornblau SM, Keating M, Resta D, Capdeville R, Talpaz M. (2002) Treatment of Philadelphia chromosome-positive, accelerated-phase chronic myelogenous leukemia with imatinib mesilate. Clin Cancer Res 8 (7): 2167–2176 12114417

[bib12] Kattan MW, Inoue Y, Giles FJ, Talpaz M, Ozer H, Guilhot F, Zuffa E, Huber SL, Beck JR (1996) Cost-effectiveness of interferon-alpha and conventional chemotherapy in chronic myelogenous leukemia. Ann Intern Med 125: 541–548 8815752 10.7326/0003-4819-125-7-199610010-00002

[bib13] Kolibaba KS, Druker BJ (2000) Current status of treatment for chronic myelogenous leukaemia. Medscape Hematology-Oncology eJournal, 3, http://www.medscape.com/viewarticle/408451 (Last checked 21 May 2002)

[bib14] Lee SJ, Anasetti C, Kuntz KM, Patten J, Antin JH, Weeks JC (1998) The costs and cost-effectiveness of unrelated donor bone marrow transplantation for chronic phase chronic myelogenous leukemia. Blood 92: 4047–4052 9834208

[bib15] Liberato NL, Quaglini S, Barosi G (1997) Cost-effectiveness of interferon alfa in chronic myelogenous leukemia. J Clin Oncol 15: 2673–2682 9215840 10.1200/JCO.1997.15.7.2673

[bib16] Messori A (1998) Cost-effectiveness of interferon in chronic myeloid leukaemia: analysis of four clinical studies. Ann Oncol 9: 389–396 9636829 10.1023/a:1008212411489

[bib17] National Institute for Clinical Excellence (2001) Technical Guidance for Manufacturers and Sponsors on Making a Submission to a Technology Appraisal. London: National Institute for Clinical Excellence

[bib18] Netten A, Rees T, Harrison G (2002) Unit Costs of Health and Social Care 2001. Personal Social Services Research Unit. University of Kent: Cantebury

[bib19] O’Brien S (2000) MIMS Guide to Chronic Myeloid Leukaemia. Medical Imprint, London

[bib20] Reese SF, Wehrle E, Gathmann I (2000) A Phase III Study of STI571 Versus Interferon-alpha Combined with Cytarabine in Patients with Newly Diagnosed Previously Untreated Philadelphia Chromosone Positive Chronic Myelogenous Leukaemia in Chronic Phase (protocol). Basel: Novartis AG

[bib21] Sawyers CL (1999) Chronic myeloid leukemia. N Engl J Med 340: 1330–1340 10219069 10.1056/NEJM199904293401706

[bib22] Sawyers CL, Hochhaus A, Feldman E, Goldman JM, Miller CB, Ottmann OG, Schiffer CA, Talpaz M, Guilhot F, Deininger MW, Fischer T, O’Brien SG, Stone RM, Gambacorti-Passerini CB, Russell NH, Reiffers JJ, Shea TC, Chapuis B, Coutre S, Tura S, Morra E, Larson RA, Saven A, Peschel C, Gratwohl A, Mandelli F, Ben-Am M, Gathmann I, Capdeville R, Paquette RL, Druker BJ (2002) Imatinib induces hematologic and cytogenic responses in patients with chronic myelogenous leukemia in myeloid blast crisis: results of a phase II study. Blood 99 (10): 3530–3539 11986204 10.1182/blood.v99.10.3530

[bib23] Smithies A (1996) Alpha Interferon in the Treatment of Chronic Myeloid Leukaemia. Southampton: Development and Evaluation Committee, Wessex Institute of Public Health Medicine

[bib24] Sonnenberg FA, Beck JR (1993) Markov models in medical decision making: a practical guide. Med Decis Making 13: 322–338 8246705 10.1177/0272989X9301300409

[bib25] The Chartered Institute of Public Finance and Accountancy (CIPFA) (2000) The Health Service Financial Database and Comparative Tool, Croydon: Institute of Public Finance Ltd

[bib26] The Italian Cooperative Study Group on Chronic Myeloid Leukemia (1994) Interferon alfa-2a as compared with conventional chemotherapy for the treatment of chronic myeloid leukemia. N Engl J Med 330: 820–825 8114834 10.1056/NEJM199403243301204

